# Occult clavicle osteomyelitis caused by *Cutibacterium acnes* (C. acnes) after coracoclavicular ligament reconstruction: A case report and review of the literature

**DOI:** 10.1016/j.ijscr.2022.107114

**Published:** 2022-04-21

**Authors:** Frederic Washburn, Britni Tran, Thomas Golden

**Affiliations:** Community Memorial Health System, 147 N. Brent St., Ventura, 93003, United States of America

**Keywords:** Clavicle osteomyelitis, Coracoclavicular reconstruction, Cutibacterium acnes, Case report

## Abstract

**Introduction:**

Clavicle osteomyelitis is a rare, but serious complication following operative repair of acromioclavicular (AC) joint separations. *Cutibacterium Acnes* (*C. acnes*) is rarely a causative pathogen in clavicle osteomyelitis and diagnosis can be challenging due to the indolent nature of this organism.

**Case presentation:**

A 45–50 year old female with a chronic AC joint separation underwent an open coracoclavicular reconstruction using semitendinosus allograft and FiberTape (Arthrex, Naples, FL). At the six month follow up, the patient complained of mild persistent pain. Imaging demonstrated widening of the medial suture tunnel with distal clavicle osteolysis, which was concerning for osteomyelitis. This was successfully treated with implant removal revision surgery and additional intravenous antibiotics. Cultures of the graft material were positive for *C. acnes.* The patient continued to have osteolysis of the distal left clavicle for a period of time despite resolution of osteomyelitis.

**Discussion:**

*C. acnes* osteomyelitis of the clavicle is difficult to diagnose because of its vague associated symptoms when implicated in infections. There are no known obvious predisposing factors for *C. acnes* clavicle osteomyelitis. Literature suggests management should include aggressive irrigation and debridement, removal of any hardware, and extended intravenous antibiotic administration.

**Conclusion:**

*C. acnes* clavicular osteomyelitis is uncommon, thus established treatment guidelines have not yet been formed. Revision surgery to remove graft material, irrigate, and debride in addition to antibiotic treatment was successful for our patient. Additional pathologic manifestations of *C. acnes* infections could include continued clavicular erosion post-clearance of infection, although further investigation is necessary.

## Highlights

• C. acnes osteomyelitis after coracoclavicular ligament reconstruction is rare.
• Culturing and identifying indolent *C. acnes* infection is difficult.
• Symptoms are generally nonspecific with unremarkable host inflammatory responses.
• All suture and graft material must be removed to eradicate this infection.
• Patient had continued distal clavicle osteolysis despite resolution of osteomyelitis.

## 1 Introduction

The acromioclavicular (AC) joint is a diarthrodial joint consisting of the distal clavicle and medial aspect of the anterior acromion [1]. AC joint injury is commonly diagnosed after acute shoulder trauma and is quite common among the athletic population [2]. When surgical intervention is required to repair AC injuries, post-operative complications such as clavicle fracture, nonunion, hardware malfunction or infection can occur [3], [4], [5]. Fractures and hardware malfunctions are the most common complications, while osteomyelitis of the clavicle is rare, but serious [2]. Common bacteria implicated in cases of clavicle osteomyelitis have been *Escherischia coli* (*E. coli*)*, Pseudomonas aeruginosa* (*P. aeruginosa*)*, methicillin resistant staphylococcus aureus* (*MRSA*)*,* and *Klebsiella pneumoniae* (*K. pneumoniae*) [6], [7].

*Cutibacterium Acnes* (*C. acnes*) (formerly *Propionibacterium Acnes*) is rarely a causative pathogen in clavicle osteomyelitis and is found in normal skin flora of the axilla, face, neck and upper back, thus making it more commonly implicated in acne vulgaris and post-operative glenohumeral infections instead [8]. There are currently no established guidelines for managing *C. acnes* clavicle osteomyelitis because of the limited number of known cases and ambiguous clinical presentations [4], [8], [9], [10], [11], [12]. Our case report details diagnosis and treatment of an indolent *C. acnes* clavicle infection post-operative repair of the AC joint. This will add to the existing literature and may shape future management of *C. acnes* clavicle osteomyelitis.

This case report is compliant with SCARE 2020 guidelines [13]. This patient was managed in a community based academic institution.

## 2 Case presentation

A 45–50 year old healthy, left-hand dominant female with no known allergies presented with a three year history of left shoulder pain after falling onto it while playing a sport. The patient has a history of hypothyroidism adequately managed with oral thyroid hormone replacement. The patient's symptoms included weakness with overhead activity as well as some pain with abduction, which was affecting day-to-day activities and lifestyle. This was initially being managed conservatively by the patient's primary care provider, but the pain and dysfunction persisted despite activity modification, physical therapy, and anti-inflammatory medication. Imaging modalities included direct bilateral anteroposterior radiographs to compare displacement against the contralateral side and magnetic resonance imaging (MRI) of the left clavicle. These demonstrated a grade III AC separation, with 1 cm of superior displacement of the left clavicle, which was measured as the distance from the top of the coracoid to the bottom of the clavicle. Given the lack of clinical improvement after extensive conservative management, the patient was referred to our clinic and it was decided a coracoclavicular (CC) reconstruction would be the best option to alleviate pain and improve function.

**Fig. 1:**
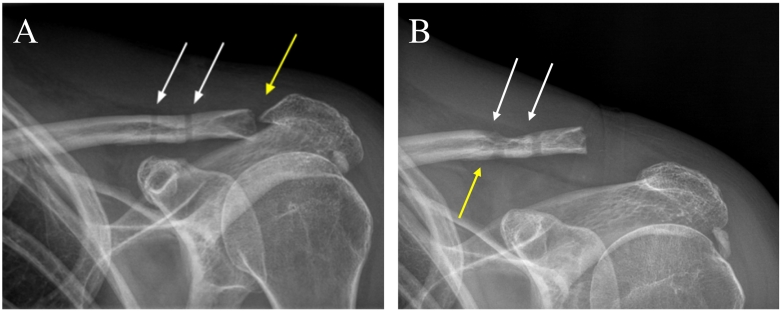
Image A: Immediate postoperative anteroposterior radiograph of the left clavicle demonstrating the coracoclavicular ligament reconstruction (white arrows) and Mumford procedure (yellow arrow). The clavicle is reduced without radiographic evidence of residual superior displacement status post procedure. Image B: Anteroposterior radiograph of the left clavicle at six month follow up appointment showing the medial and lateral suture tunnels (white arrows) with widening of the medial suture tunnel (yellow arrow) and slight superior displacement of the clavicle. (For interpretation of the references to colour in this figure legend, the reader is referred to the web version of this article.)

The patient was placed under general anesthesia and positioned supine and reclined at a 45 degree angle in the “beach chair” position. The left shoulder was subsequently draped and sterilized. Through an open technique, the CC ligament reconstruction was performed using a semitendinosus allograft looped around the coracoid process and tied over the top of the clavicle. This was reinforced by FiberTape (Arthrex, Naples, FL) around the coracoid process and through separate drill holes in the anatomic position of the trapezoid and conoid ligaments. Findings during surgery confirmed arthrosis of the AC joint and a Mumford procedure was performed as well (Fig. 1A). The patient tolerated the procedure well and was placed in a shoulder abduction sling postoperatively.

The patient returned to the outpatient clinic a week later for postoperative follow-up and reported persistent pain and low grade fevers since discharge from the hospital. Physical examination revealed slight serous drainage and mild erythema surrounding the wound. No significant swelling or evidence of purulent drainage was noted. At this time, cultures were taken and the patient was admitted to the hospital for suspected overlying cellulitis to be treated with intravenous Vancomycin 750 mg. Initial serological tests showed a white blood cell count (WBC) of 10,500/mcl, c-reactive protein (CRP) of 1.5-mg/dL, and erythrocyte sedimentation rate (ESR) of 34-mm/h. The patient responded well to intravenous Vancomycin and had improvement of inflammatory markers after treatment. Cultures during this admission demonstrated no growth to date. An infectious disease specialist was consulted prior to the patient's discharge and a plan for a single injection of Orbactiv 1200 mg was agreed upon, as the patient declined outpatient intravenous antibiotic therapy. The patient was discharged from the hospital with marked improvement of all symptoms.

The subsequent follow ups over the next six months in the outpatient clinic showed no significant changes, so further recommendations included physical therapy and continued home exercise. At the patient's six month appointment, she returned with mild intermittent pain over the posterolateral tip of the clavicle despite self-reported adherence to suggested physical therapy. An x-ray of the left shoulder and AC joint was ordered, showing widening of the medial suture tunnel with slight superior displacement of the clavicle (Fig. 1B). At this time, all inflammatory markers were within normal limits and physical examination showed no abnormal findings, so the patient was treated with a corticosteroid injection. She was then instructed to return in one month for a follow up and possible revision surgery to remove the suture and graft material if pain persisted.

**Fig. 2:**
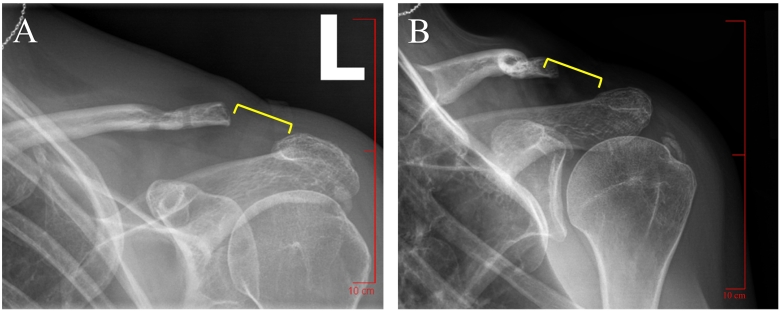
Anteroposterior (image A) and grashey (image B) radiographs demonstrating distal left clavicular erosion (yellow bracket) prior to second revision surgery for graft material removal, subacromial decompression, calcium debridement, and arthroscopy. (For interpretation of the references to colour in this figure legend, the reader is referred to the web version of this article.)

At the patient's one month follow up appointment, persistent pain was reported and distal clavicular erosion was appreciated on radiographs, which was concerning for clavicular osteomyelitis (Fig. 2). Due to persistent pain and worsening x-ray findings, it was agreed that arthroscopy, subacromial decompression, debridement of calcium and an open modified Mumford procedure along with the removal of suture and graft material were indicated. The surgical procedure was performed by the same orthopedic surgeon who initially reconstructed the patient's CC ligament. The patient was placed under general anesthesia and positioned in a right lateral decubitus position. The left shoulder was sterilized and draped, in addition to having eight-pounds of suspension applied. The prominent distal 5 mm of clavicle was excised and as much of the suture and graft material was removed as possible. The patient tolerated the revision procedure well without complications. Chronic inflammation tissue was noted intraoperatively, but no purulence was appreciated. Tissue specimens demonstrated no growth on cultures, but cultures of the graft and suture fixations demonstrated growth of *C. acnes*. The patient was given a 10-day course of oral 500 mg Duricef and a four week course of parenteral Vancomycin via a peripherally inserted central catheter (PICC) line for treatment of osteomyelitis secondary to *C. acnes* infection.

**Fig. 3:**
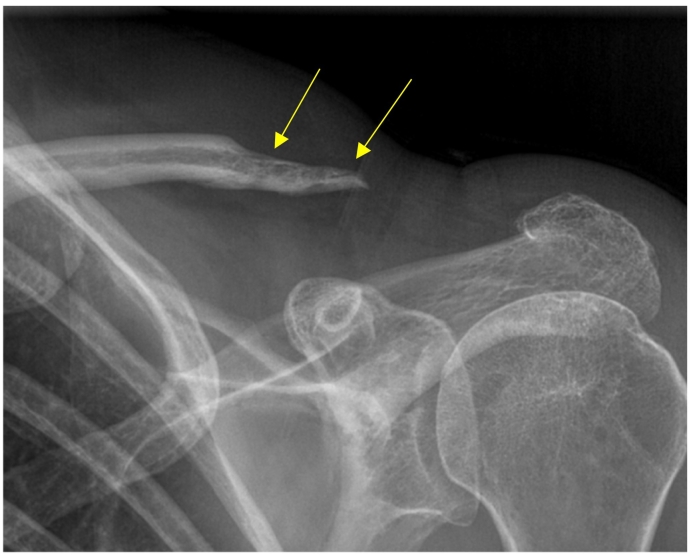
One month anteroposterior follow-up radiograph demonstrating continued osteolysis (yellow arrows) despite resolution of symptoms and no remaining limitations in function status-post revision surgery. (For interpretation of the references to colour in this figure legend, the reader is referred to the web version of this article.)

**Fig. 4:**
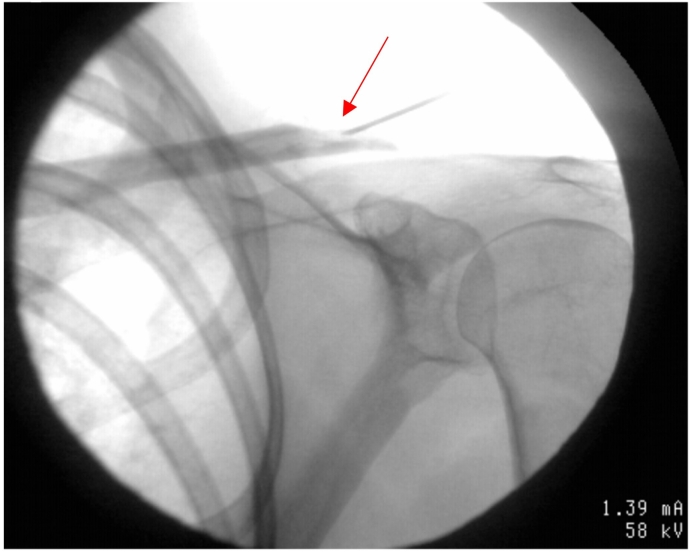
Fluoroscopically assisted needle biopsy (red arrow) after finding of continued distal left clavicle osteolysis after revision surgery, removal of implants, and four week course of intravenous Vancomycin. MRI demonstrated resolution of osteomyelitis. Cultures of the biopsy demonstrated no growth of *C. acnes* three weeks after. (For interpretation of the references to colour in this figure legend, the reader is referred to the web version of this article.)

**Fig. 5:**
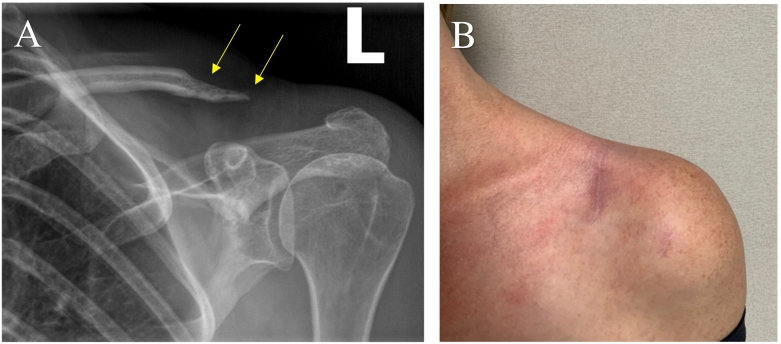
Four month anteroposterior follow-up radiograph (Image A) showing eventual cessation and stability of osteolysis (yellow arrows) while the patient remains asymptomatic with no functional deficits. Clinical photo (Image B) demonstrating no obvious clinical deformity with healed surgical incisions. (For interpretation of the references to colour in this figure legend, the reader is referred to the web version of this article.)

One month after revision surgery, the patient was doing exceptionally well, with no pain or limitations in function. However, a repeat x-ray showed further osteolysis of the distal clavicle (Fig. 3). An MRI was ordered which demonstrated no findings suggestive of osteomyelitis. The continued osteolysis after revision surgery was still a concerning and inexplicable finding, and definitive confirmation that the patient's clavicular osteomyelitis had completely resolved was warranted. A fluoroscopically-assisted needle biopsy was performed, which demonstrated no growth of *C. acnes* at three weeks (Fig. 4). A recheck x-ray several months later showed no further progression of the distal clavicle osteolysis. The patient reported resolution of all symptoms and no functional deficits. Physical exam showed no visible deformities with well-healed surgical incisions (Fig. 5). The patient continues to be monitored routinely in our clinic, but there has not been any further osteolysis and she remains functionally intact with a good prognosis.

## 3 Discussion

*C. acnes* is a facultatively anaerobic, gram positive rod shaped bacteria that is commonly found as both a contaminant and causative pathogen in post-operative glenohumeral infections, but the incidence of *C. acnes* clavicle infections appears to be much less [8], [10], [14], [15]. When this organism is suspected, multiple reports have emphasized repeated and exhaustive microbiologic investigation must be performed to culture this fastidious organism [8], [14], [16], [17]. A review of literature revealed limited cases regarding clavicle osteomyelitis due to bacteria such as *E. coli*, *P. aeruginosa*, *K. pneumoniae*, and *MRSA* [6], [7]. Even fewer cases showed *C. acnes* clavicle osteomyelitis; two cases occurred spontaneously via presumed hematogenous spread without prior trauma, surgery, instrumentation, or injections, while other case reports showed *C. acnes* infections and chronic nonunion after failed surgical open reduction and surgical fixations (ORIF) [4], [5], [9], [11]. Our case presentation is an addition to the growing literature that demonstrates clavicular osteomyelitis with *C. acnes*, while uncommon, is important and should be carefully considered in post-operative care.

The underlying commonality between these cases, including our own, was difficulty culturing and diagnosing *C. acnes* infections due to the slow-growing and indolent nature of this microbe. There is still large amount of debate over whether positive *C. acnes* cultures are sometimes an “orthopedic red herring” or simply “commensal” incidental findings, which contributes to much of the confusion and lack of standardized treatment protocol for *C. acnes* osteomyelitis [8], [10], [11], [12]. Further adding to the ambiguity, symptoms of *C. acnes* infections are generally nonspecific and do not follow typical host inflammatory responses such as swelling, erythema drainage, tenderness or sinus tracts [9], [10]. Inflammatory lab markers such as ESR, CRP and WBC are also commonly unchanged [4], [8], [11]. If classic signs of infection do present, it is usually quite slow in onset and can even have no clinical manifestations for up to two years [8], [10].

Once diagnosed, treatment of *C. acnes* clavicular osteomyelitis was similar despite no established “gold standard” protocol. Aggressive irrigation and debridement, hardware removal (if any), and extended IV antibiotic administration resolved the infection [4], [5], [8], [9], [11]. First line treatment for deep *C. acnes* infections in particular were commonly intravenous Vancomycin or Clindamycin for six to eight weeks, but it should be noted that *C. acnes* has conferred resistance to at least one type of broad spectrum antibiotic [5], [6], [9], [11]. Resistance to penicillins, tetracyclines, rifampicin, and erythromycin is thought to be secondary to poor compliance and experimental use of broad spectrum antibiotic treatment for acne vulgaris associated with *C. acnes* [11]. *C. acnes*' growing antimicrobial resistance not only makes it difficult to treat, but contributes to persistent osteomyelitis [11]. This warrants additional treatment options such as excision of the distal clavicle or muscle flap placement over the affected area [4], [5], [6].

The prolonged and insidious *C. acnes* infection in our patient's case was likely due to three reasons. First, *C. acnes* biofilm production provides resistance against host immunity and antimicrobial treatment [18]. Secondly, *c. acnes* growth was demonstrated in the suture material, and the indolent infection was only able to be eradicated once all of the suture was removed. Third, our patient should have received IV Vancomycin for at least six to eight weeks, but was instead given a single injection dose of 1200 mg Orbactiv per her request upon discharge [5], [6], [9], [11]. These factors may have contributed to the indolent positive *C. acnes* culture causing osteomyelitis six months later that may not have been resolved completely when she was initially admitted for overlying cellulitis. Additionally, our patient had a period of continued distal clavicle erosion in the months following osteomyelitis resolution despite multiple negative cultures. The erosion eventually ceased, but this presentation was not described in any other literature to date and could be further investigated as a possible consequence of *C. acnes* clavicle osteomyelitis.

## 4 Conclusion

*C. acnes* is a fastidious organism that is difficult to diagnose and treat for a multitude of reasons. Our case report describes similar findings and treatment modalities as other clavicular osteomyelitis literature, but the notable differences between each case such as our patient's continued distal clavicle osteolysis demonstrates there is still much to be investigated about this rising orthopedic pathogen. A high index of suspicion in addition to proper and meticulous microbiological techniques are essential to culture and diagnose *C. acnes* infections, especially in instances when patients display vague symptoms with unremarkable inflammatory markers following shoulder girdle operative procedures. There are currently no established clinical guidelines for treatment and management of *C. acnes* osteomyelitis. Further studies should be conducted to determine the best course of action to efficiently and effectively prevent and resolve future infections.

## Funding

None.

## Sources of funding

This research did not receive any specific grant from funding agencies in the public, commercial, or not-for-profit sectors.

## Ethical approval

This case report was conducted with the approval of the Institutional Review Boards.

## Consent

Written informed consent was obtained from the patient for publication of this case report and accompanying images. A copy of the written consent is available for review by the Editor-in-Chief of this journal on request.

## Research registration

None.

## Guarantor

Dr. Frederic Washburn, D.O.

## Provenance and peer review

Not commissioned, externally peer-reviewed.

## CRediT authorship contribution statement

Dr. Frederic Washburn, D.O.: supervision, writing, critical review and final approval of the article.

Britni Tran, B.S.: writing, critical revisions.

Dr. Thomas Golden, M.D.: study conception, supervision, critical review and final approval of the article.

Accountability for all aspects of work: all authors.

## Declaration of competing interest

All authors declare no conflicts of interest.
